# Bromhexine hydrochloride enhances the therapeutic efficacy of tiamulin against experimental *Staphylococcus aureus* infection in dogs: targeting bacterial virulence, boosting antioxidant defense, and improving histopathology

**DOI:** 10.3389/fphar.2025.1679854

**Published:** 2025-12-18

**Authors:** Hanem F. El-Gendy, Shimaa R. Masoud, Nagwa I. Sheraiba, Shimaa S. Elnahriry, Doaa A. Madkour, Reda M. S. Korany, Hazim O. Khalifa, Hanaa Y. Elnagar

**Affiliations:** 1 Department of Pharmacology, Faculty of Veterinary Medicine, University of Sadat City, Sadat City, Egypt; 2 Department of Physiology, Faculty of Veterinary Medicine, University of Sadat City, Sadat City, Egypt; 3 Department of Husbandry and Animal Wealth Development, Faculty of Veterinary Medicine, University of Sadat City, Sadat City, Egypt; 4 Department of Bacteriology, Mycology and Immunology, Faculty of Veterinary Medicine, University of Sadat City, Sadat City, Egypt; 5 Department of Biochemistry and Chemistry of Nutrition, Faculty of Veterinary Medicine, University of Sadat City, Sadat City, Egypt; 6 Department of Pathology, Faculty of Veterinary Medicine, Cairo University, Cairo, Egypt; 7 Department of Pathology, Faculty of Veterinary Medicine, Egyptian Chinese University, Cairo, Egypt; 8 Department of Veterinary Medicine, College of Food and Agriculture, United Arab Emirates University, Al Ain, United Arab Emirates; 9 Cytology and Histology Department, Faculty of Veterinary Medicine, University of Sadat City, Sadat City, Egypt

**Keywords:** antioxidant, bromhexine, dogs, efficacy, inflammatory cytokine, *Staphylococcus aureus*, tiamulin, virulence

## Abstract

**Introduction:**

*Staphylococcus aureus* is a prominent pathogen capable of causing systemic infections and multi-organ damage, primarily driven by its high virulence and induction of oxidative stress. This study evaluated the therapeutic efficacy of tiamulin alone and in combination with bromhexine in a canine model of systemic *S. aureus* infection, focusing on oxidative stress biomarkers, bacterial burden, tissue histopathology, and the expression of cardiac and bacterial virulence-related genes.

**Methods:**

Experimental infection was induced in dogs, except for a healthy control group. Animals were assigned to five groups: uninfected control, infected untreated, tiamulin-treated, bromhexine-treated, and tiamulin plus bromhexine-treated. Oxidative stress was assessed through measurements of malondialdehyde (MDA) and total antioxidant capacity (TAC) in cardiac, hepatic, and renal tissues. Bacterial load was quantified, and minimum inhibitory concentrations (MICs) of the treatments were determined. Quantitative PCR was performed to evaluate the expression of *S. aureus* virulence genes including *hla* (alpha-hemolysin), *ebpS* (extracellular matrix-binding protein S), and *icaA* (intercellular adhesion A). Histopathological analyses of heart, liver, and kidney tissues were conducted, and hematological and biochemical parameters (total protein, albumin, and globulin) were measured. Cardiac injury markers, cytochrome P450 1B1 (*CYP1B1*) and interleukin-1 beta (*IL-1β*), were also assessed.

**Results:**

Infected untreated animals exhibited significantly elevated MDA, decreased TAC, high bacterial loads, severe histopathological alterations, and upregulated expression of *IL-1β* and *CYP1B1*. Tiamulin monotherapy produced moderate reductions in oxidative stress and bacterial burden. The combination of tiamulin and bromhexine resulted in a significant reduction in MDA, restoration of TAC, lower MIC values, suppressed expression of virulence genes (p < 0.05), and near-normal tissue architecture. Cardiac gene expression analysis showed substantial downregulation of *IL-1β* and *CYP1B1* in the combination-treated group, indicating alleviation of inflammation and cardiac injury.

**Discussion:**

The combination therapy of tiamulin and bromhexine exhibited superior protective effects against *S. aureus* infection compared to monotherapy or untreated infection. These benefits appear to be mediated through synergistic antimicrobial, anti-virulence, and antioxidant mechanisms. The findings support the potential of this therapeutic approach for managing drug-resistant *S. aureus* infections and justify further clinical investigation.

## Graphical Abstract

**Figure:**
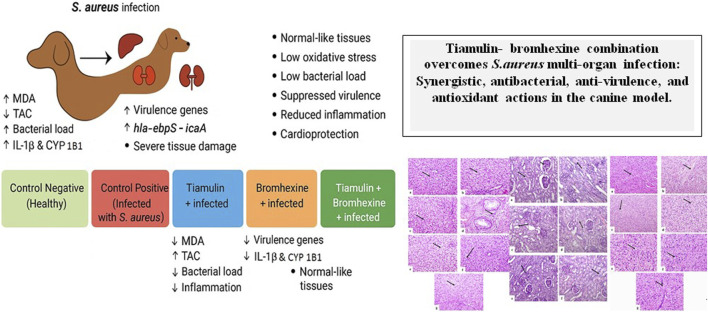

## 1 Introduction

The rapid emergence of multidrug-resistant (MDR) bacterial infections represents a significant threat to the efficacy of current antibiotic therapies and constitutes a major global public health challenge (Soliman et al., 2016). In response, the World Health Organization (WHO) has prioritized a group of critical MDR pathogens—including *Staphylococcus aureus*, *Enterococcus faecium*, *Klebsiella pneumoniae*, *Pseudomonas aeruginosa*, *Acinetobacter baumannii*, and *Enterobacter* spp.—as leading causative agents of healthcare-associated infections. This alarming situation underscores the urgent need to develop innovative antimicrobial strategies to counteract MDR bacterial diseases (Ahmed et al., 2015).

Among these pathogens, *S. aureus* stands out as a significant zoonotic agent with the capacity to survive under diverse and extreme environmental conditions (Khalifa et al., 2024). It has developed resistance to multiple classes of antibiotics, notably methicillin, resulting in methicillin-resistant *S. aureus* (MRSA), which exhibits broad resistance to most β-lactam antibiotics (Khalifa et al., 2024; Oreiby et al., 2019). In addition to its antibiotic resistance, *S. aureus* readily forms biofilms, further enhancing its resilience against antimicrobial agents and complicating clinical treatment. This pathogen’s ability to infect various organs—including the heart, lungs, bones, brain, liver, and kidneys—contributes to its high virulence and adaptability, causing conditions that range from mild dermal infections to life-threatening systemic diseases (Mustafa et al., 2022; Touaitia et al., 2025).

Combining mucolytic agents with antibiotics has emerged as a promising approach to enhance therapeutic outcomes in MDR bacterial infections (Sui et al., 2025). Although studies on the concurrent use of bromhexine and tiamulin against *S. aureus* infections in dogs are limited, the pharmacodynamic properties of both drugs suggest potential synergistic effects. Bromhexine’s mucolytic action may improve antibiotic penetration into infected tissues, particularly in cases of respiratory tract infections, such as pulmonary pneumonia and septicemia caused by *S. aureus* (Parker and Prince, 2012).

Tiamulin is a semisynthetic pleuromutilin derivative, originating from the natural diterpene compound isolated from *Pleurotus mutilus* in 1951. Its unique tricyclic structure enables specific binding to the 50S ribosomal subunit of bacteria, thereby inhibiting protein synthesis. Tiamulin exhibits potent bacteriostatic activity against *Mycoplasma* spp., Gram-positive bacteria, and *Leptospira* spp., with possible bactericidal effects at higher concentrations. It is approved solely for veterinary use by the European Medicines Agency (EMA) and the U.S. Food and Drug Administration (FDA) (Zhou et al., 2023; Sartini et al., 2023). Nonetheless, its administration must be carefully controlled due to the risk of adverse effects, including hepatotoxicity, nephrotoxicity, neurotoxicity, and cardiotoxicity (Pankowska et al., 2024).

Bromhexine, a widely used mucolytic agent, is a synthetic derivative of vasicine—an alkaloid extracted from the medicinal plant *Adhatoda vasica* (Ahmadi et al., 2024). It reduces mucus viscosity and enhances mucociliary clearance, thereby facilitating deeper tissue penetration of co-administered antibiotics and improving their pharmacological efficacy (Deretic and Timmins, 2019). Additionally, bromhexine exhibits anti-inflammatory properties, which may help alleviate respiratory tract inflammation and contribute indirectly to cardiovascular protection (Marinov et al., 2025).

This study aims to investigate the therapeutic efficacy of bromhexine and tiamulin, individually and in combination, in the treatment of *S. aureus* infections in dogs. It further seeks to determine whether bromhexine can potentiate the antimicrobial activity of tiamulin, particularly in infections associated with potential cardiotoxic effects.

## 2 Materials and methods

### 2.1 Chemicals and bacterial isolates

Tiamulin hydrogen fumarate (purity ≥98.0%) was obtained in powder form from Pharma-Swede (Cairo, Egypt). Bromhexine hydrochloride (98% purity) was also kindly provided by Pharma-Swede® as a white crystalline powder; its chemical name is 2-amino-3,5-dibromobenzyl methylamine hydrochloride. The *Staphylococcus aureus* strain ATCC 6538 used in this study was procured from the Animal Health Research Institute, Shibin Elkom branch, Egypt. This strain exhibited key virulence traits, including catalase positivity and the presence of virulence-associated genes. In addition to conventional biochemical identification (Supplementary Table 1), the identity of the *S. aureus* strain was molecularly confirmed by polymerase chain reaction (PCR) following the method described by Mohammed et al. (2025).

### 2.2 Determination of minimum inhibitory concentration (MIC) against *S. aureus*

The minimum inhibitory concentrations (MICs) of tiamulin, bromhexine hydrochloride, and their combination against *Staphylococcus aureus* strain ATCC 6538 were determined *in vitro*. A bacterial suspension of *S. aureus* at a concentration of 10^7^ CFU/mL was prepared in its specific culture medium. Tiamulin was serially diluted two-fold across a concentration range of 0.7812–100 μg/mL using the culture medium as a 1:1 diluent. Similarly, bromhexine hydrochloride was subjected to two-fold serial dilutions ranging from 16 to 2048 μg/mL.

Combination assays were also conducted using fixed-ratio dilutions of the two agents. All experiments were performed in triplicate to ensure reproducibility and accuracy. The MIC was defined as the lowest concentration of tiamulin, bromhexine, or their combination that inhibited visible bacterial growth, as indicated by the absence of a color change in the culture medium. It should be noted that the fractional inhibitory concentration index (FICI) was not calculated in this study, as the combination assay was designed primarily to determine MIC changes rather than to quantify synergistic effects (Abonashey et al., 2024).

### 2.3 *In vitro* assessment of *S. aureus* virulence-related gene expression level using PCR and real-time PCR

#### 2.3.1 Genotypic and gene expression analysis

Genotypic characterization of *Staphylococcus aureus* isolates was performed to detect key virulence-associated genes, including *hla* (alpha-hemolysin), *ebpS* (extracellular matrix-binding protein S), and *icaA* (intercellular adhesion gene A). The 16S small subunit ribosomal RNA gene (*16S rRNA*) was used as a housekeeping gene. Primers used in this study, supplied by Metabion (Germany), are listed in Supplementary Table 3. Previous research has demonstrated that exposure to sub-MIC levels of antibiotics, including tiamulin and bromhexine, can modulate the expression of virulence genes such as *hla*, *ebpS*, and *icaA* within 3 hours (Yang et al., 2020). Conventional PCR primers targeting these genes were validated for use in SYBR Green-based quantitative PCR (qPCR), as endpoint PCR primers have been shown to be effective for real-time PCR applications (Thornton and Basu, 2011).

#### 2.3.2 DNA extraction and PCR

Genomic DNA was extracted using the QIAamp DNA Mini Kit (Qiagen, Germany, GmbH) with minor modifications to the manufacturer’s protocol. Briefly, 200 µL of bacterial suspension was incubated with 20 µL of proteinase K and 200 µL of lysis buffer at 56 °C for 10 min. Following incubation, 200 µL of absolute ethanol was added, and the sample was processed according to the standard washing and centrifugation steps. DNA was eluted in 100 µL of the elution buffer provided in the kit. Uniplex PCR was performed in 25 µL reaction volumes containing 12.5 µL of EmeraldAmp Max PCR Master Mix (Takara, Japan), 1 µL of each primer (20 pmol), 5.5 µL of nuclease-free water, and 5 µL of DNA template. Amplifications were conducted in an Applied Biosystems 2,720 thermal cycler. PCR products were resolved on 1.5% agarose gels (Applichem, Germany, GmbH) using 1× TBE buffer at room temperature (5 V/cm). For analysis, 20 µL of uniplex and 30 µL of multiplex PCR products were loaded per well. A GelPilot 100 bp Plus DNA Ladder (Qiagen, GmbH, Germany) was used to estimate fragment sizes. Gels were visualized using a gel documentation system (Alpha Innotech, Biometra), and band analysis was performed with dedicated software.

#### 2.3.3 RNA extraction and quantitative real-time PCR (qPCR)

To prevent RNA degradation, 1 mL of RNAprotect Bacteria Reagent (Qiagen, Germany, GmbH) was added to 0.5 mL of harvested bacterial culture. The mixture was vortexed, incubated at room temperature for 5 min, and centrifuged at 8,000 rpm for 10 min. The resulting pellet was resuspended in 200 µL of Tris-EDTA buffer containing 1 mg/mL lysozyme (Biochemica, Applichem), followed by the addition of 700 µL of RLT buffer supplemented with 10 μL/mL β-mercaptoethanol. Subsequently, 500 µL of 100% ethanol was added, and total RNA was extracted following the Enzymatic Lysis of Bacteria protocol provided in the QIAamp RNeasy Mini Kit (Qiagen, Germany, GmbH).

The one-step SYBR Green RT-qPCR reaction mixture (25 µL total volume) included 12.5 µL of 2× QuantiTect SYBR Green PCR Master Mix (Qiagen, Germany, GmbH), 0.25 µL of RevertAid Reverse Transcriptase (200 U/µL, Thermo Fisher), 0.5 µL of each primer (20 pmol), 8.25 µL of nuclease-free water, and 3 µL of RNA template. Amplification was carried out in a Stratagene MX3005P real-time PCR system under the following thermal profile: reverse transcription at 50 °C for 30 min; initial denaturation at 94 °C for 15 min; followed by 40 cycles of 94 °C for 15 s, annealing at 49 °C–55 °C (depending on the primer) for 30 s, and extension at 72 °C for 30 s. The dissociation (melting) curve protocol included 95 °C for 1 min and a stepwise increase from 65 °C (+0.5 °C per cycle) for 61 cycles to confirm specificity of the amplified products. Amplification curves and threshold cycle (Ct) values were analyzed using the Stratagene MX3005P software. To evaluate changes in gene expression, Ct values for each gene in treated samples were compared to those in the positive control group using the 2^–ΔΔCt^ method described by Yuan et al. (2006). Fold changes were calculated to determine upregulation or downregulation of *hla*, *ebpS*, and *icaA* in response to treatment with sub-MIC levels of tiamulin and bromhexine.

### 2.4 Animals and trial design

**FIGURE 1:**
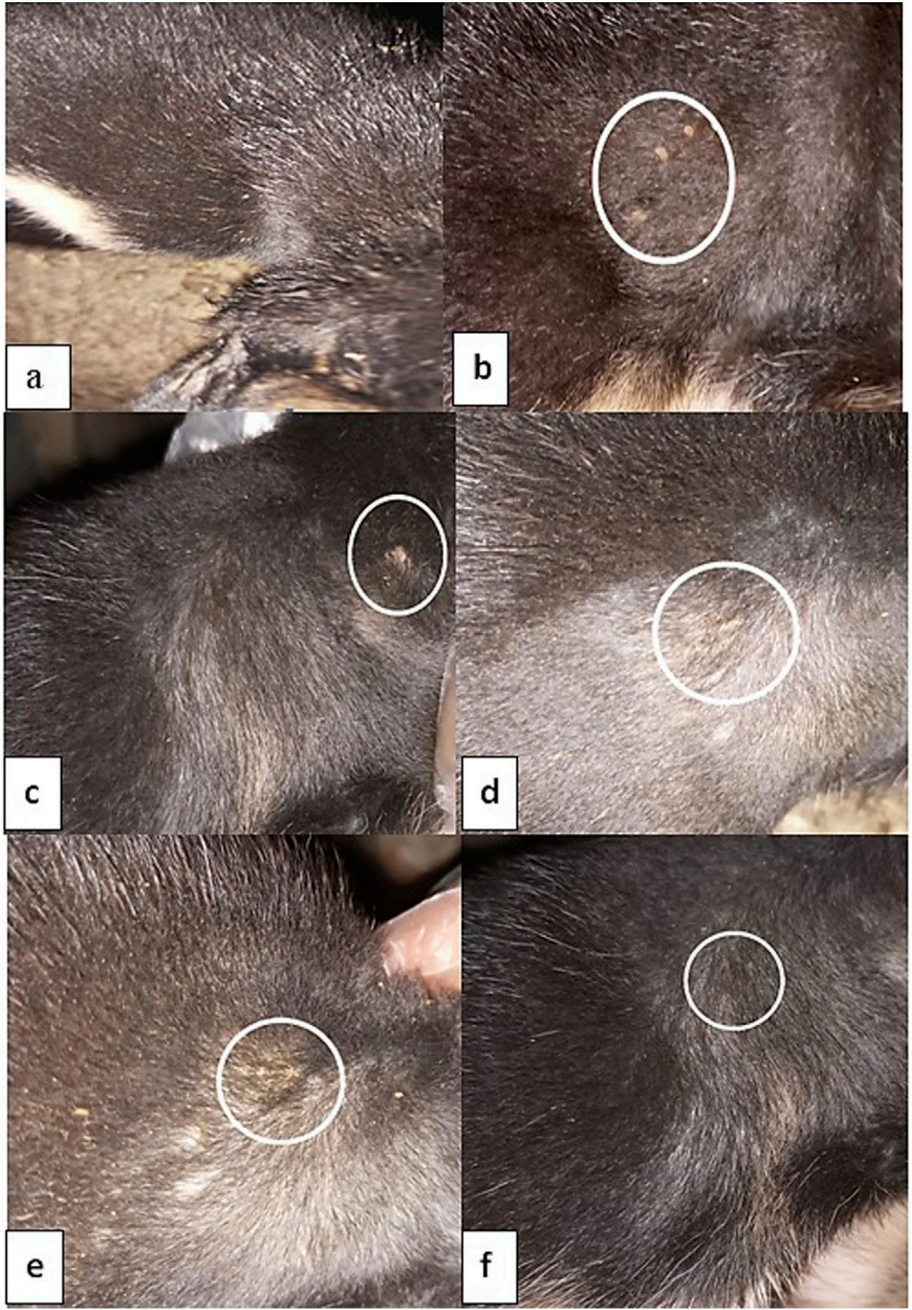
Representative images showing the development and healing of skin lesions in *Staphylococcus aureus*–infected dogs. **(a)** Normal uninfected skin; **(b,c)** infected, untreated group displaying visible redness, swelling, and crust formation; **(d)** tiamulin-treated group showing partial recovery and reduced inflammation; **(e)** bromhexine-treated group showing moderate improvement with decreased erythema and crusting; **(f)** combination-treated group (tiamulin + bromhexine) exhibiting near-complete healing and hair regrowth, indicating enhanced therapeutic efficacy. Lesion severity was scored as follows: 0 = normal skin; 1 = mild erythema or minimal crusts; 2 = moderate erythema with visible crusts or partial hair loss; 3 = severe lesions with ulceration, thick crusts, or extensive inflammation. Representative scores: **(a)** 0, **(b,c)** 2, **(d)** 1, **(e)** 1–2, **(f)** 0–1.

A total of twenty-five male dogs, between 3 and 6 months of age, were individually housed for a 2-week acclimation period, during which they had unrestricted access to food and water. The local Egyptian dog breed, commonly referred to as 'Baladi dogs', was used in this study. These dogs were obtained from a trusted local farm specializing in native breeds, El Monoufiya, Egypt. The owners' informed consent was obtained for the participation of their animals in this study. All experimental procedures complied with the Guidelines for Animal Experimentation and received approval from the Ethical Committee of the Faculty of Veterinary Medicine, University of Sadat City, Egypt. Animal care and handling were performed in line with the regulations and standards of the Animal Care House (Approval No. VUSC-007-1-25). The potential effect of bromhexine hydrochloride on the efficacy of tiamulin against *Staphylococcus aureus* infection in dogs was determined using a protocol similar to that previously described by Radi et al. (2020). Dogs were randomly allocated into five groups of five dogs each, as illustrated in Supplementary Table 2. After shaving each dog’s fur, the skin was cleaned with soap and sterile water. Dogs were given a 1 mL intradermal inoculation under local anesthesia with 10^5^ *S. aureus* (strain ATCC 6538) colony-forming units, as previously reported by Kamr et al. (2020) and Arbaga et al. (2021), except the negative control group, which was injected with 1 mL of normal saline. The onset of cutaneous lesions occurred 3 days after injection. Skin infection was induced in the experimental animals according to standardized procedures. The successful establishment of the infection model was confirmed by observing characteristic clinical signs, including erythema (redness), edema (swelling), pus formation, and necrotic tissue at the site of inoculation within 48–72 h post-infection. Representative photographic images of the lesions were captured at different time points (pre-infection, post-infection, and post-treatment) to document the progression of infection and the therapeutic effects of the treatments, including the combination therapy. These images provide visual confirmation of model establishment and wound healing outcomes as in Figure 1. On day 3 post-inoculation, the skin lesions were swabbed using sterile swabs to verify their authenticity. To identify the developed colonies, samples were cultivated on Baird-Parker agar medium at 37 °C for 24 h after being subjected to Gram staining. Dogs were separated into groups as shown in Supplementary Table 2. The experiment lasted for 9 days.

Group 1, the negative control group, was intradermally injected with normal saline on day 1 and received 1 mL of distilled water orally for five consecutive days starting on day 3. In Group 2, the positive control group, dogs were injected intradermally with 1 mL of 10^5^ CFU *S. aureus* on day 1 and received 1 mL of distilled water orally for five consecutive days starting on day 3. Group 3 dogs were injected with 1 mL of 10^5^ CFU *S. aureus* on day 1 and, beginning on day 3, received tiamulin at a dose of 10 mg/kg body weight once daily (every 24 h) orally for 5 days (Laber, 1988; Garmyn et al., 2017). Group 4 dogs were injected with 1 mL of 10^5^ CFU *S. aureus* on day 1 and, starting on day 3, received bromhexine hydrochloride at a dose of 1 mg/kg body weight twice daily (every 12 h) orally for 5 days (Radi et al., 2020). Group 5 dogs were injected with 1 mL of 10^5^ CFU *S. aureus* on day 1 and, beginning on day 3, received tiamulin (10 mg/kg body weight, once daily) along with bromhexine hydrochloride (1 mg/kg body weight, twice daily) orally for five consecutive days.

### 2.5 Quantitative assessment of *S. aureus* in treated and control groups

Skin swab samples were aseptically collected from pyoderma lesions of dogs in each group 5 days after the initiation of treatment using sterile cotton swabs. The swabs were gently rotated over the infected area to ensure adequate sampling while avoiding contamination from surrounding healthy skin. Each swab was transferred into a sterile tube containing phosphate-buffered saline (PBS), vortexed, and subjected to serial tenfold dilutions. From the 10^3^ and 10^5^ dilutions, 100 µL aliquots were inoculated onto Baird-Parker Agar (Oxoid, United Kingdom) plates and incubated aerobically at 37 °C for 24–48 h. Presumptive *S*. *aureus* colonies appeared as black, shiny colonies with clear halos, measuring 1–3 mm in diameter with distinct margins. Colonies were confirmed through Gram staining, catalase, and coagulase tests. Bacterial counts were expressed as colony-forming units per milliliter (CFU/mL). Isolates were identified using conventional microbiological techniques, including Gram staining and a series of biochemical assays such as coagulase activity, catalase test, mannitol fermentation, urease activity, nitrate reduction, mannose and lactose fermentation, phosphatase, arginine dihydrolase, and indoxyl phosphatase activity, as outlined in Supplementary Table 1 (Koneman et al., 2006).

### 2.6 Blood and tissue sampling

On the ninth day, following an overnight fast, blood samples were collected from the jugular vein of each dog. Each sample was divided into two aliquots: one treated with EDTA for hematological analysis, and the other left untreated to allow clotting. The clotted samples were centrifuged at 3,000 rpm for 15 min to separate the serum, which was subsequently stored at −20 °C for biochemical assays. After blood collection, the animals were euthanized *via* intravenous administration of a lethal dose of pentobarbital. Tissue specimens from the heart, liver, and kidneys were harvested (Tao et al., 2022). A portion of each tissue was stored at −80 °C for subsequent biochemical and gene expression analyses, while the remaining portions were fixed in 10% neutral buffered formalin for histopathological examination.

### 2.7 Hematological analysis

Immediately after collection, blood samples were subjected to hematological analysis to determine total leukocyte count (TLC), differential leukocyte counts, hemoglobin concentration (Hb), platelet count (Plt), hematocrit percentage (PCV%), and red blood cell count (RBCs), using an automated hematology analyzer (Sysmex F-800, Tokyo, Japan) (El-Gendy et al., 2024a).

### 2.8 Biochemical assay

Biochemical markers related to cardiac, hepatic, and renal function were assessed in the collected serum samples, following the manufacturers' protocols. For cardiac evaluation, total creatine kinase (CK) and creatine kinase-MB (CK-MB), specific to myocardial tissue, were measured using an ELISA kit (CK-MB, ThermoFisher Scientific, Waltham, MA, USA), as described by Aujla and Patel (2022). Additionally, troponin I levels were quantified using a commercial ELISA kit (Troponin, ThermoFisher Scientific, Waltham, MA, USA), according to the method outlined by Jiang et al. (2017), in accordance with the manufacturer’s instructions. For hepatic evaluation, hepatic enzymes, including alanine aminotransferase (ALT) and aspartate aminotransferase (AST), were measured in serum samples using commercially available kits supplied by Biodiagnostic Company (Dokki, Giza, Egypt) (El-Gendy et al., 2022). For renal evaluation, renal biomarkers were evaluated in serum samples using commercial kits in accordance with the manufacturer’s instructions (Biomed Company, Cairo, Egypt). Renal function indicators, including creatinine, blood urea nitrogen (BUN), and urea, were quantified as previously described (Li et al., 2024). Additionally, serum total protein, albumin, and globulin concentrations were determined following the method of Henry et al. (1974).

### 2.9 Absolute and relative organ weights

On the day of sacrifice, the dogs were weighed using a precision scale. Following euthanasia, the heart, liver, and kidneys were carefully excised, cleared of surrounding tissues, and weighed. The relative organ weights (ROW) were calculated according to the method described by Mazukina et al. (2025), using the following formula: ROW = [Absolute organ weight (g)/Body weight of dog (g)] × 100.

### 2.10 Assessment of oxidative and antioxidant biomarkers in tissue homogenates

Portions of hepatic, renal, and cardiac tissues were homogenized in cold phosphate-buffered saline (PBS) using a glass homogenizer to prepare 25% (w/v) tissue homogenates. The homogenates were centrifuged at 10,000–12,000 rpm for 5 min, and the resulting supernatants were stored at −80 °C for subsequent analyses. Oxidative stress markers and antioxidant capacity were assessed colorimetrically using commercial assay kits (Biodiagnostic, Dokki, Giza, Egypt), following the manufacturer’s instructions. Specifically, malondialdehyde (MDA) levels were measured as described by Bahramibanan et al. (2025), and total antioxidant capacity (TAC) was evaluated according to Cordiano et al. (2023).

### 2.11 Assessment of cardiac gene expression level using quantitative RT-PCR

RNA was extracted from cardiac tissue samples using the Direct-zol™ RNA Miniprep Plus Kit (Zymo Research, Cat# R2072, USA), following the manufacturer’s protocol. This method allows direct purification of high-quality total RNA, including small RNAs, from *TRIzol®*-treated samples using Zymo-Spin™ IIICG columns. On-column DNase I treatment was performed to eliminate residual genomic DNA. RNA yield and purity were assessed spectrophotometrically (Beckman, USA) by measuring absorbance at 260 nm and calculating the A260/A280 ratio.

Subsequently, reverse transcription and real-time PCR (RT-qPCR) were performed using the SuperScript™ IV One-Step RT-PCR System (Thermo Fisher Scientific, Cat# 12594100, USA), which enables cDNA synthesis and amplification in a single tube using gene-specific primers. Reactions were conducted on a StepOne™ Real-Time PCR System (Applied Biosystems, USA). Specific primers for inflammation, mitochondrial dysfunction, and cardiac injury marker genes (Supplementary Table 3) were verified for specificity using NCBI BLAST.

The thermal cycling conditions were as follows: reverse transcription at 55 °C for 10 min; enzyme inactivation at 95 °C for 2 min; followed by 40 cycles of denaturation at 95 °C for 10 s, annealing at 55 °C for 10 s, and extension at 72 °C for 30 s.

Gene expression levels were evaluated using cycle threshold (Ct) values. The Ct values of target genes (*IL-1β* and *CYP1B1*) were normalized to the reference gene (*GAPDH*). A control group was used as a calibrator to assess relative expression. Relative quantification (RQ) was calculated using the 2^−ΔΔCT^ method (Livak and Schmittgen, 2001), allowing determination of fold changes in gene expression.

### 2.12 Histopathological examination

Liver, kidney, and heart tissue samples were collected and promptly fixed in 10% neutral buffered formalin. Following fixation, the tissues were washed thoroughly, dehydrated through a graded series of alcohol, cleared in xylene, and embedded in paraffin wax. Sections of 5 μm thickness were then prepared from the paraffin blocks and stained with hematoxylin and eosin (H&E) for histological examination, according to the standard method outlined by Bancroft and Gamble (2008). Histopathological alterations in liver, kidney, and heart were scored as: no changes (0), mild (1), moderate (2), and severe (3) changes. The grading was determined by percentage: <30% changes (mild change), 30%–50% (moderate change), and >50% (severe change) (Zaki et al., 2024).

### 2.13 Statistical analysis

The data were analyzed using one-way ANOVA (SPSS, version 21). Duncan’s multiple range test was applied to compare group means at a significance level of *p* < 0.05. The statistical procedures followed the guidelines of Sendecor and Cochran (1956). Results are expressed as means ± standard error (SE).

## 3 Results

### 3.1 Comparative analysis of bacterial load (CFU/mL) among study groups

**FIGURE 2:**
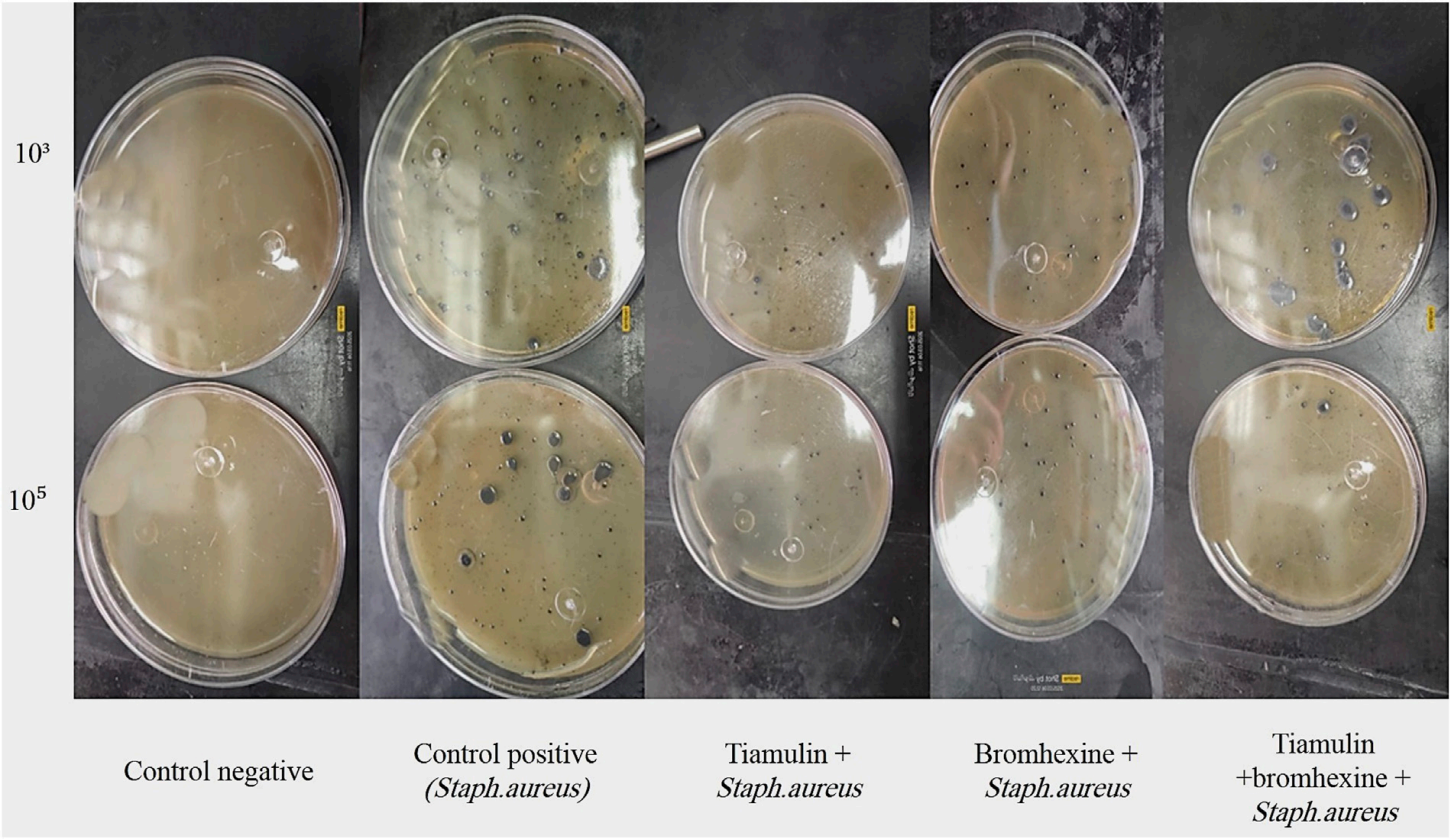
Bacterial count (CFU/mL) in different treatment groups at 10^3^ and 10^5^ dilutions.

Table 1 and Figure 2 present the quantitative evaluation of bacterial loads (CFU/mL) across the experimental groups. All treatment groups exhibited a notable and statistically significant reduction in bacterial counts compared to the positive control group (G2), at both 10^3^ and 10^5^ dilution levels. This consistent decrease underscores the potent antibacterial activity of the tested interventions.

**TABLE 1:** Comparison of bacterial Counts (CFU/mL) between tiamulin monotherapy and tiamulin–bromhexine combination therapy.

Groups	G1	G2	G3	G4	G5	P-value
10^3^ dilutions	5.00 ± 0.73^a^	65.00 ± 2.55^b^	23.17 ± 2.38^c^	39.66 ± 0.92^d^	30.00 ± 3.58^e^	0.002
10^5^ dilutions	1.50 ± 0.22^c^	43.66 ± 2.23^b^	10.00 ± 1.34^e^	22.00 ± 0.45^d^	13.33 ± 0.56^e^	0.606

^a^ Means with no shared superscript in the same row vary significantly (*p* < 0.05).

^b^ Means with no shared superscript in the same row vary significantly (*p* < 0.05).

^c^ Means with no shared superscript in the same row vary significantly (*p* < 0.05).

^d^ Means with no shared superscript in the same row vary significantly (*p* < 0.05).

^e^ Means with no shared superscript in the same row vary significantly (*p* < 0.05).

Notably, the group treated with the combined therapy of bromhexine and tiamulin (Group 5) demonstrated substantial bacterial suppression. Although the reduction in CFU/ml at the 10^5^ dilution level was not statistically significant compared to the tiamulin-only group, the values remained closely comparable, suggesting a similar therapeutic effect. These findings indicate that co-administration of bromhexine does not compromise the antibacterial efficacy of tiamulin and may potentially enhance it, possibly through improved drug delivery or mucolytic action. Overall, statistical analysis confirmed significant differences among the groups (*P* < 0.05), supporting the antibacterial effectiveness of the applied treatment protocols.

### 3.2 *In vitro* MIC values

The *in vitro* MICs of the isolated *Staphylococcus aureus* strain were determined using the broth microdilution method and are presented in Supplementary Table 4. The MIC values for tiamulin, bromhexine, and their combination (tiamulin + bromhexine) were 12.5, 128, and (6.25 + 64) μg/mL, respectively. These findings indicate that the tested treatments effectively reduced bacterial resistance *in vitro*. Notably, the combination therapy demonstrated an improved MIC compared to either agent alone, suggesting a potential synergistic or additive effect.

### 3.3 Molecular detection of virulence genes by PCR and RT- PCR

The presence of selected virulence-associated genes in the *Staphylococcus aureus* isolates was confirmed by conventional PCR. Amplification products corresponding to the *hla*, *icaA*, and *ebpS* genes were successfully detected by agarose gel electrophoresis, with distinct bands appearing at their respective expected sizes: *hla* (704 bp), *icaA* (1,315 bp), and *ebpS* (652 bp), as shown in Supplementary Figure 2. Additionally, the 16S rRNA gene (∼1,500 bp) was consistently amplified in all samples, confirming bacterial identity and serving as a positive internal control.

Furthermore, real-time PCR using SYBR Green fluorescence detection was employed to assess the expression levels of selected *S. aureus* virulence genes. Consistent amplification curves and specific melt peaks confirmed the reliability of this method, supporting the gel-based findings (Table 2; Supplementary Figure 3, *p* < 0.05). Relative gene expression analysis revealed a significant reduction in the expression of virulence genes in samples treated with the combination of bromhexine and tiamulin, followed by bromhexine alone and tiamulin alone, respectively.

**TABLE 2:** *In vitro* quantitative detection of virulence targeted genes in *S. aureus* isolates.

Genes	*16S rRNA*	*Hla*	*ebpS*	*ica A*	P-value
Control (*S. aureus)*	1.00 ± 0.001	1.00 ± 0.001^a^	1.00 ± 0.001^a^	1.00 ± 0.001^a^	0.0001
Tiamulin + *S. aureus*	1.00 ± 0.001	0.44 ± 0.006^b^	0.61 ± 0.0005^b^	0.48 ± 0.003^b^	0.0001
Bromhexine + *S. aureus*	1.00 ± 0.001	0.25 ± 0.003^c^	0.36 ± 0.0016^c^	0.36 ± 0.003^c^	0.0001
Tiamulin + Bromhexine + *S. aureus*	1.00 ± 0.001	0.07 ± 0.006^d^	0.21 ± 0.0005^d^	0.10 ± 0.0005^d^	0.0001

16S rRNA, 16S small subunit ribosomal RNA, gene; hla, alpha-hemolysin gene; ebpS, extracellular matrix-binding protein S gene; ica A, intercellular adhesion gene A.

^a^ Means with no shared superscript in the same column vary significantly (*p* < 0.05).

^b^ Means with no shared superscript in the same column vary significantly (*p* < 0.05).

^c^ Means with no shared superscript in the same column vary significantly (*p* < 0.05).

^d^ Means with no shared superscript in the same column vary significantly (*p* < 0.05).

### 3.4 Absolute and relative organ weights

In our experiments, absolute organ weights varied considerably among the groups, reflecting corresponding differences in total body weight. Significant differences were observed in both absolute and relative weights of the liver, kidneys, and heart across the treatment groups, as presented in Table 3. Specifically, relative liver and kidney weights were significantly higher than those of the control group (*p* < 0.05). Additionally, the relative heart weight was significantly increased in Groups 3 and 5 compared to the other groups (*p* < 0.05).

**TABLE 3:** Effect of tiamulin and bromhexine on absolute and relative organs weight of dogs infected with *S. aureus*.

Groups parameters	G1	G2	G3	G4	G5	P-value
Absolute liver weight (g)	156.67 ± 1.52^a^	178.47 ± 0.18^b^	165.03 ± 2.74^c^	162.90 ± 0.95^c^	171.83 ± 0.57^d^	0.004
Relative liver weight (%)	9.55 ± 0.09^c^	13.06 ± 0.11^b^	10.11 ± 0.15^d^	10.40 ± 0.12^d^	9.61 ± 0.10^c^	0.0001
Absolute kidney weight (g)	22.63 ± 0.23^c^	23.46 ± 0.18^c^ ^,^ ^d^	24.16 ± 0.33^b^ ^,^ ^d^	25.03 ± 0.73^b^	22.60 ± 0.22^c^	0.001
Relative kidney weight (%)	1.38 ± 0.02^a^	1.71 ± 0.02^b^	1.48 ± 0.01^c^	1.59 ± 0.05^d^	1.26 ± 0.02^e^	0.003
Absolute heart weight (g)	21.60 ± 0.20^e^	25.36 ± 0.20^a^	36.06 ± 0.40^d^	27.66 ± 0.55^c^	42.50 ± 0.76^b^	0.0001
Relative heart weight (%)	1.31 ± 0.02^a^	1.85 ± 0.02^c^	2.21 ± 0.02^d^	1.76 ± 0.04^c^	2.37 ± 0.04^b^	0.0001

^a^ Means with no shared superscript in the same row vary significantly (*p* < 0.05).

^b^ Means with no shared superscript in the same row vary significantly (*p* < 0.05).

^c^ Means with no shared superscript in the same row vary significantly (*p* < 0.05).

^d^ Means with no shared superscript in the same row vary significantly (*p* < 0.05).

^e^ Means with no shared superscript in the same row vary significantly (*p* < 0.05).

### 3.5 Blood measurements

Table 4 presents the effects of tiamulin, bromhexine, and their combination on erythrogram parameters in dogs experimentally infected with *S. aureus*. Compared to the infected untreated group (G2), all treated groups (G3–G5) showed a significant restoration of packed cell volume (PCV) to near-normal levels (*p* < 0.05). Mean corpuscular volume (MCV) was highest in G3 (75.06 ± 0.02) and lowest in G1 (64.26 ± 0.22), with significant differences observed among groups bearing different superscript letters (*p* < 0.05). Regarding mean corpuscular hemoglobin (MCH), G3 exhibited the highest value (25.62 ± 0.04), which was significantly greater than all other groups (*p* < 0.05), while no significant differences were noted among the remaining groups. For mean corpuscular hemoglobin concentration (MCHC), significantly higher values were observed in G1, G3, G4, and G5 compared to G2 (*p* < 0.05). Overall, the infected untreated dogs (G2) demonstrated significantly lower erythrogram indices than the treated groups (*p* < 0.05). Platelet counts were significantly higher in G1 (459 ± 14.45) compared to all other groups, which showed marked reductions in platelet levels (*p* < 0.05). Moreover, G2 exhibited significantly elevated total leukocyte counts (TLC), neutrophil percentages, and neutrophil-to-lymphocyte (N/L) ratio, along with a significant reduction in lymphocyte percentages, when compared to the treated groups (*p* < 0.05).

**TABLE 4:** Effect of Tiamulin and bromhexine on blood profile of dogs.

Groups parameters	G1	G2	G3	G4	G5	P-value
Hb (g/dL)	9.22 ± 0.04^a^	8.02 ± 0.14^b^	9.06 ± 0.02^a^	9.72 ± 0.17^c^	8.66 ± 0.11^d^	0.012
RBCs (10^6^/μL)	3.78 ± 0.012^c^	3.01 ± 0.14^d^	3.45 ± 0.01^a^	3.86 ± 0.02^c^	3.48 ± 0.002^a^	0.027
PCV (%)	24.24 ± 0.02^a^	23.86 ± 0.11^d^	25.76 ± 0.02^a^	28.16 ± 1.61^c^	25.76 ± 0.02^a^	0.025
MCV (fL)	64.26 ± 0.22^b^	71.52 ± 0.77^a^	75.06 ± 0.02^c^	68.40 ± 1.83^d^	74.18 ± 0.23^a^ ^,^ ^c^	0.003
MCH (pg)	22.66 ± 0.02^a^	23.30 ± 0.78^a^	25.62 ± 0.04^c^	23.36 ± 0.26^a^	23.89 ± 0.68^a^	0.004
MCHC (g/dL)	35.02 ± 0.04^c^	32.52 ± 0.77^d^	34.22 ± 0.04^a^ ^,^ ^c^	34.08 ± 0.41^a^ ^,^ ^c^	33.42 ± 0.22^a^	0.0001
PLTs(×10^3^/μL)	459 ± 14.45^c^	174 ± 0.83^a^ ^,^ ^d^	165 ± 0.48^a^ ^,^ ^d^	245 ± 7.89^a^	131 ± 0.97^d^	0.004
TLC (10^3^/μL)	12.06 ± 0.02^e^	18.50 ± 0.22^c^	17.68 ± 0.07^a^	15.18 ± 0.07^b^	17.08 ± 0.18^d^	0.0001
Neutrophil (%)	19.00 ± 0.002^a^ ^,^ ^d^	33.60 ± 4.63^c^	23.40 ± 0.24^a^	11.00 ± 0.001^b^	14.60 ± 0.24^b^ ^,^ ^d^	0.0001
Lymphocyte (%)	65.40 ± 0.24^a^ ^,^ ^c^	52.40 ± 4.69^a^	61.60 ± 0.24^a^ ^,^ ^d^	58.80 ± 0.20^d^	70.60 ± 0.24^c^	0.020
Monocyte (%)	10.60 ± 0.24^a^	10.40 ± 0.24^a^	10.00 ± 0.001^a^ ^,^ ^d^	26.20 ± 0.20^c^	9.40 ± 0.24^d^	0.001
Eosinophil (%)	4.00 ± 0.0001^c^	2.60 ± 0.24^a^	4.00 ± 0.001^c^	3.00 ± 0.002^a^	4.40 ± 0.24^c^	0.024
Basophil (%)	1.00 ± 0.001	1.00 ± 0.001	1.00 ± 0.001	1.00 ± 0.001	1.00 ± 0.001	0.06
N/L ratio	0.29 ± 0.001^a^	0.69 ± 0.14^c^	0.38 ± 0.005^a^	0.18 ± 0.0006^a^	0.20 ± 0.002^a^	0.003

RBCs, red blood cell count; Hb, hemoglobin; PCV, packed cell volume; MCV, mean corpuscular volume; MCH, mean corpuscular hemoglobin; MCHC, mean corpuscular hemoglobin concentration; PLTs, platelets; TLC, total leukocyte count; N/L ratio, neutrophil-to-lymphocyte ratio.

^a^ Values are expressed as mean ± standard error (SE), with a sample size of n = 5. Means within the same row that do not share a common superscript letter differ significantly (p < 0.05).

^b^ Values are expressed as mean ± standard error (SE), with a sample size of n = 5. Means within the same row that do not share a common superscript letter differ significantly (p < 0.05).

^c^ Values are expressed as mean ± standard error (SE), with a sample size of n = 5. Means within the same row that do not share a common superscript letter differ significantly (p < 0.05).

^d^ Values are expressed as mean ± standard error (SE), with a sample size of n = 5. Means within the same row that do not share a common superscript letter differ significantly (p < 0.05).

^e^ Values are expressed as mean ± standard error (SE), with a sample size of n = 5. Means within the same row that do not share a common superscript letter differ significantly (p < 0.05).

### 3.6 Serum biochemical markers in experimentally *S. aureus* -infected dogs

Cardiac, renal, and hepatic serum markers are presented in Table 5. A significant increase in CK and CK-MB levels was observed in the infected untreated group (G2) compared to the healthy control group (G1) and other treatment groups (*P* < 0.05). Troponin I levels showed no significant differences among the groups. Serum ALT and AST levels, indicators of hepatic enzyme activity, were significantly elevated in G2 compared to G1 (*P* < 0.05). Notably, G5 demonstrated a numerical reduction in both ALT and AST levels compared to G3 and G4. Additionally, G4 and G5 exhibited significantly reduced total protein and albumin levels relative to the infected untreated group (G2), while no significant differences were observed in globulin levels and A/G ratio among groups (*P* < 0.05). Renal function markers—including creatinine, urea, and blood urea nitrogen (BUN)—were also assessed. The infected untreated group (G2) showed a significant increase in all three parameters compared to G1 and the treatment groups (*P* < 0.05). The combination treatment group (G5) displayed the most pronounced improvements, with substantial reductions across most parameters.

**TABLE 5:** Effect of treatments on serum biochemical markers in experimentally *S. aureus*-infected dogs.

Groups parameters	G1	G2	G3	G4	G5	P-value
CK (U/L)	1,669 ± 1.18^a^ ^,^ ^b^	2,732 ± 28.00^c^	1833 ± 6.36^a^	1744 ± 0.001^a^	1,539 ± 17.58^b^	0.0001
CK-MB (ng/mL)	284 ± 12.26^b^	449 ± 17.94^c^	324 ± 4.16^a^	389 ± 0.022^a^	367 ± 14.13^a^	0.011
Troponin I (ng/mL)	0.03 ± 0.005	0.05 ± 0.003	0.05 ± 0.002	0.06 ± 0.001	0.048 ± 0.004	0.06
ALT (U/L)	17.20 ± 0.73^b^	39.80 ± 3.00^c^	35.60 ± 0.24^a^	33.40 ± 2.15^a^	26.40 ± 1.77^a^ ^,^ ^b^	0.028
AST (U/L)	37.60 ± 2.46^b^	65.60 ± 2.24^c^	55.00 ± 2.04^a^	52.80 ± 0.86^a^	47.40 ± 2.27^a^ ^,^ ^b^	0.018
Total protein (g/dL)	4.17 ± 0.06^c^	4.31 ± 0.03^c^	4.25 ± 0.02^a^ ^,^ ^c^	3.73 ± 0.07^b^	3.98 ± 0.12^a^ ^,^ ^b^	0.001
Albumin (g/dL)	2.44 ± 0.13^c^	2.36 ± 4.50^c^	2.24 ± 0.02^a^ ^,^ ^c^	1.82 ± 0.08^b^	1.97 ± 0.12^a^ ^,^ ^b^	0.001
Globulin (g/dL)	1.73 ± 0.11	1.95 ± 4.53	2.01 ± 0.04	1.91 ± 0.12	2.01 ± 0.11	0.432
A/G ratio	1.45 ± 0.16	0.79 ± 0.50	1.11 ± 0.04	0.97 ± 0.09	0.99 ± 0.09	0.354
Creatinine (mg/dL)	0.44 ± 0.012^a^	0.97 ± 0.23^c^	0.50 ± 0.005^a^	0.37 ± 0.012^a^	0.43 ± 0.025^a^	0.004
BUN (mg/dL)	7.26 ± 0.57^a^	8.89 ± 0.52^c^	7.89 ± 0.24^a^	7.01 ± 0.004^b^	7.57 ± 0.22^a^	0.014
Urea (mg/dL)	16.80 ± 0.48^a^	40.00 ± 5.18^c^	20.60 ± 1.12^a^	17.40 ± 0.81^a^	17.20 ± 0.20^a^	0.010

Values are represented as means ± SE, n = 5. CK, creatine kinase; CK-MB, creatine kinase-MB; ALT, alanine aminotransferase; AST, aspartate aminotransferase; A/G ratio, albumin/globulin ratio; BUN, blood urea nitrogen.

^a^ Means with no shared superscript in the same row vary significantly (*p* < 0.05).

^b^ Means with no shared superscript in the same row vary significantly (*p* < 0.05).

^c^ Means with no shared superscript in the same row vary significantly (*p* < 0.05).

### 3.7 Tissue oxidant and antioxidant parameters

**FIGURE 3:**
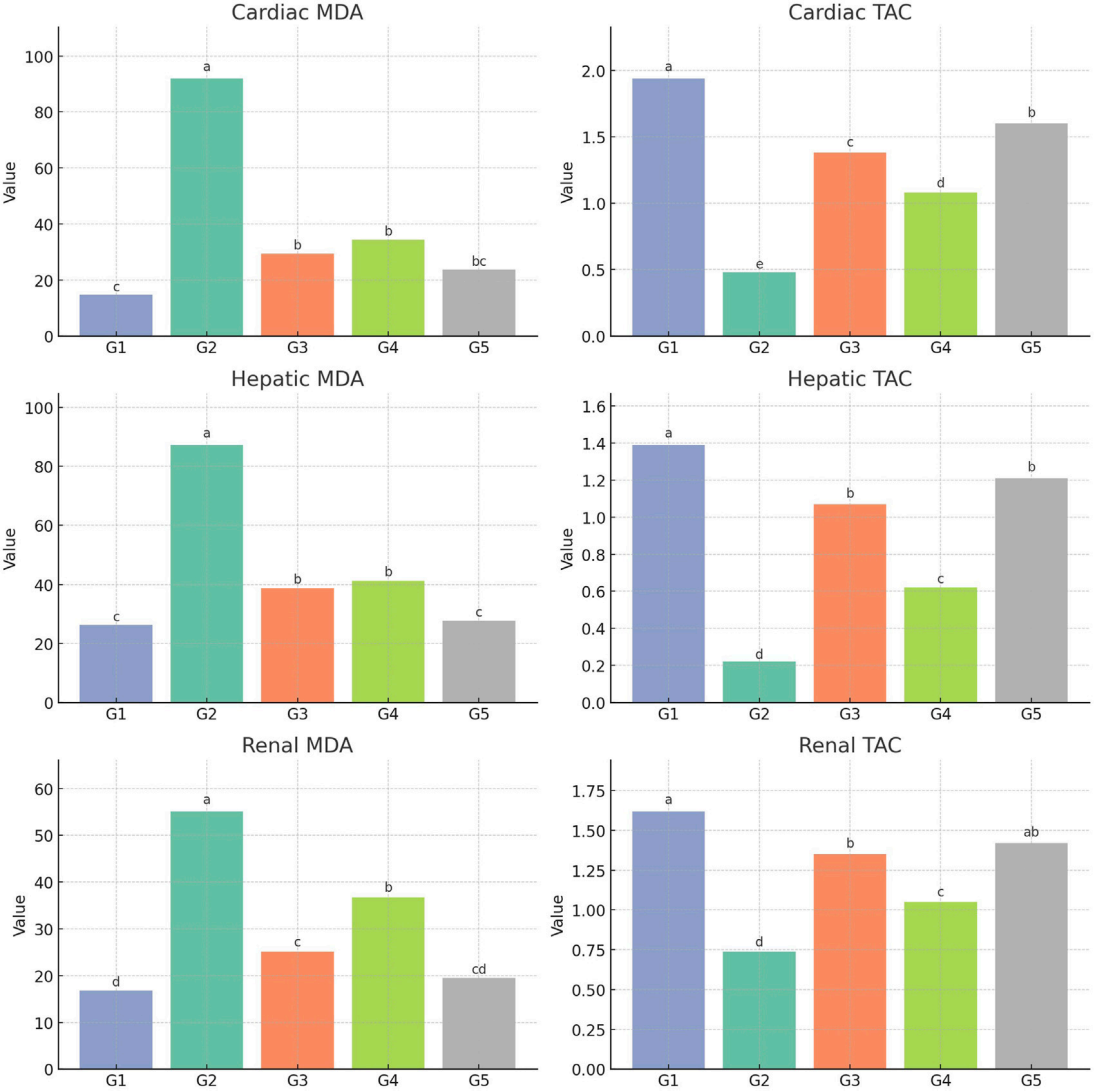
Tissue oxidant/antioxidant biomarkers, MDA; malondialdehyde, TAC; total antioxidant capacity. Values are represented as means ± SE.

The MDA levels in the heart, liver, and kidney were significantly elevated in the infected untreated group (G2) compared to the normal control group (G1) (*P* < 0.05), indicating increased oxidative stress. Treatment with either agent alone (G3 and G4) led to a significant, moderate reduction in MDA levels compared to G2. However, the combination treatment (G5) resulted in a marked and significant decrease in MDA levels, approaching near-normal values and outperforming both monotherapies (*P* < 0.05). Similarly, TAC was markedly reduced in the infected group (G2). Although both monotherapies modestly improved TAC levels, the combined treatment in G5 produced the highest TAC values, significantly exceeding the effects of either treatment alone (*P* < 0.05), suggesting enhanced antioxidant defense (Figure 3).

### 3.8 Cardiac gene expression

**FIGURE 4:**
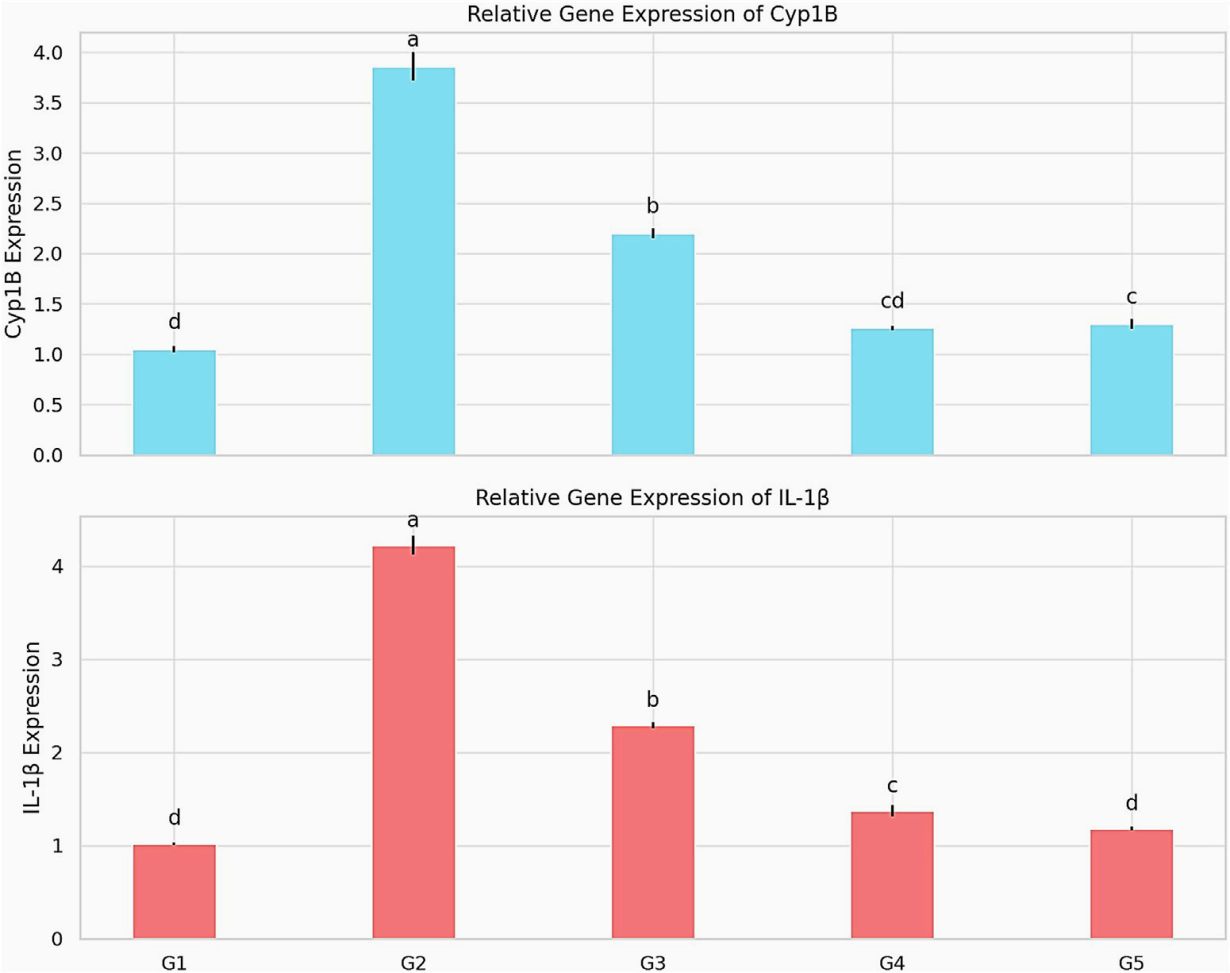
Relative expression levels of *CYP1B1* and *IL-1β* genes across experimental groups. Data are presented as mean ± SEM, with statistical significance indicated by different letters (p < 0.001). *CYP1B1*: cytochrome P450 family 1 subfamily B member 1; *IL-1β*: interleukin-1 beta.

Gene expression analysis demonstrated a significant upregulation of *IL-1β* in the infected untreated group (G2) compared to the control group (G1), indicating a pronounced inflammatory response (*P* < 0.001). Notably, all treatment groups exhibited a reduction in *IL-1β* expression, with the combination therapy group (G5) showing the most substantial decrease. Similarly, *CYB1B1* expression was significantly elevated in the infected untreated group (G2), consistent with heightened oxidative stress. Treatment—particularly in groups G4 and G5—resulted in a marked downregulation of *CYB1B1*, approaching normal expression levels. This suggests a protective role of the treatments in mitigating oxidative stress and modulating harmful metabolic processes (Figure 4).

### 3.9 Histopathological findings

**FIGURE 5:**
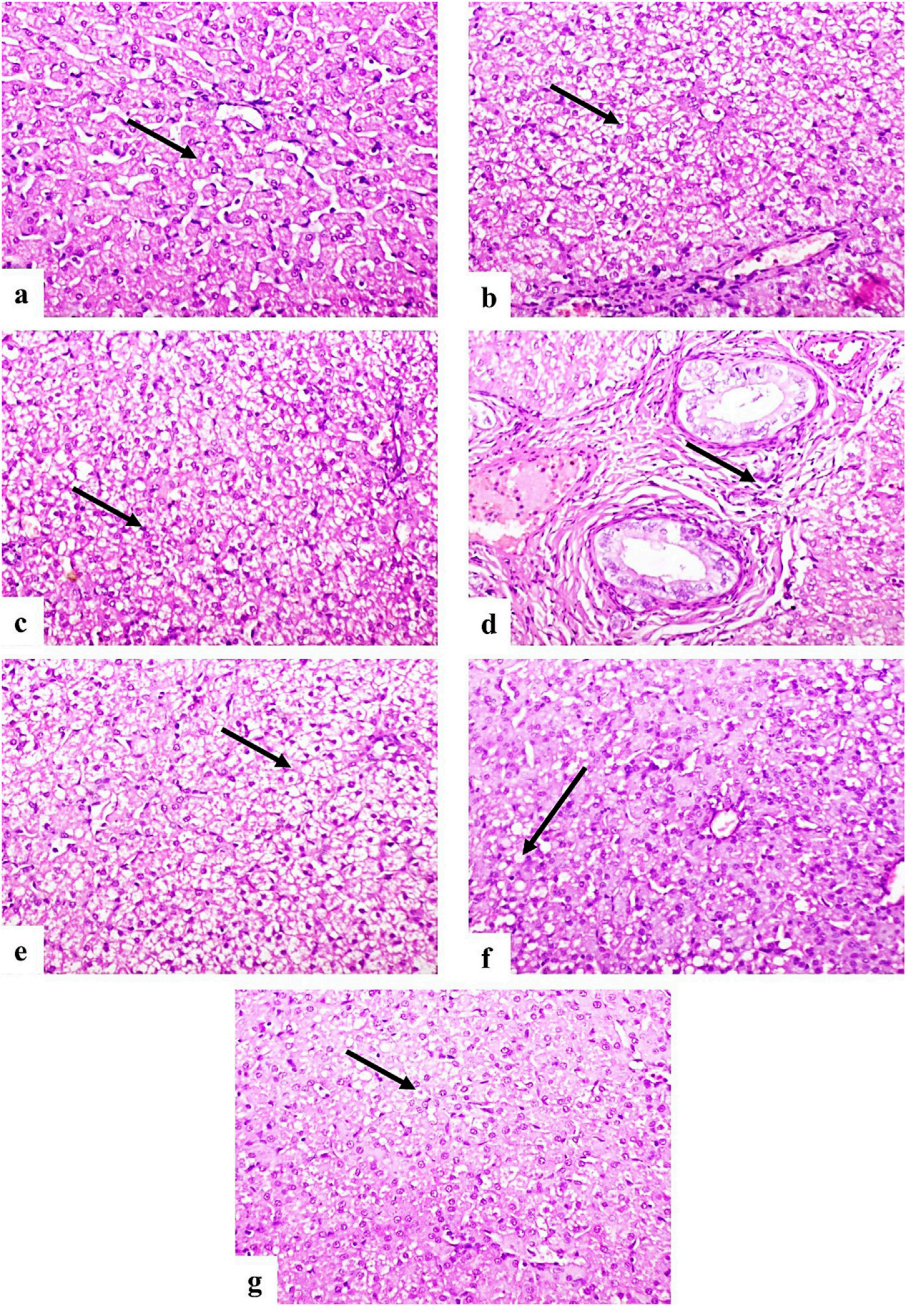
Photomicrographs of dog liver sections stained with H&E (×200). **(a)** Control group showing normal histological structure of hepatocytes (arrow). **(b–d)** G2 group showing diffuse vacuolar degeneration **(b)**, coagulative necrosis **(c)**, and portal fibrosis with mononuclear inflammatory cell infiltration **(d)** (arrows). **(e)** G3 showing moderate vacuolar degeneration and necrosis (arrow). **(f)** G4 showing mild vacuolar degeneration (arrow). **(g)** G5 showing few degenerated hepatocytes (arrow).

Histopathological examination revealed normal liver architecture in the control group (G1) (Figure 5a). In contrast, the infected untreated group (G2) showed severe, diffuse vacuolar degeneration, coagulative necrosis of hepatocytes (Figures 5b, c), and moderate fibrosis accompanied by mononuclear inflammatory cell infiltration in the portal areas (Figure 5d). Group G3 exhibited moderate vacuolar degeneration and hepatocellular necrosis (Figure 5e), while G4 showed only mild vacuolar changes in a few hepatocytes (Figure 5f). Notably, G5 presented minimal hepatic degeneration and necrosis (Figure 5g).

**FIGURE 6:**
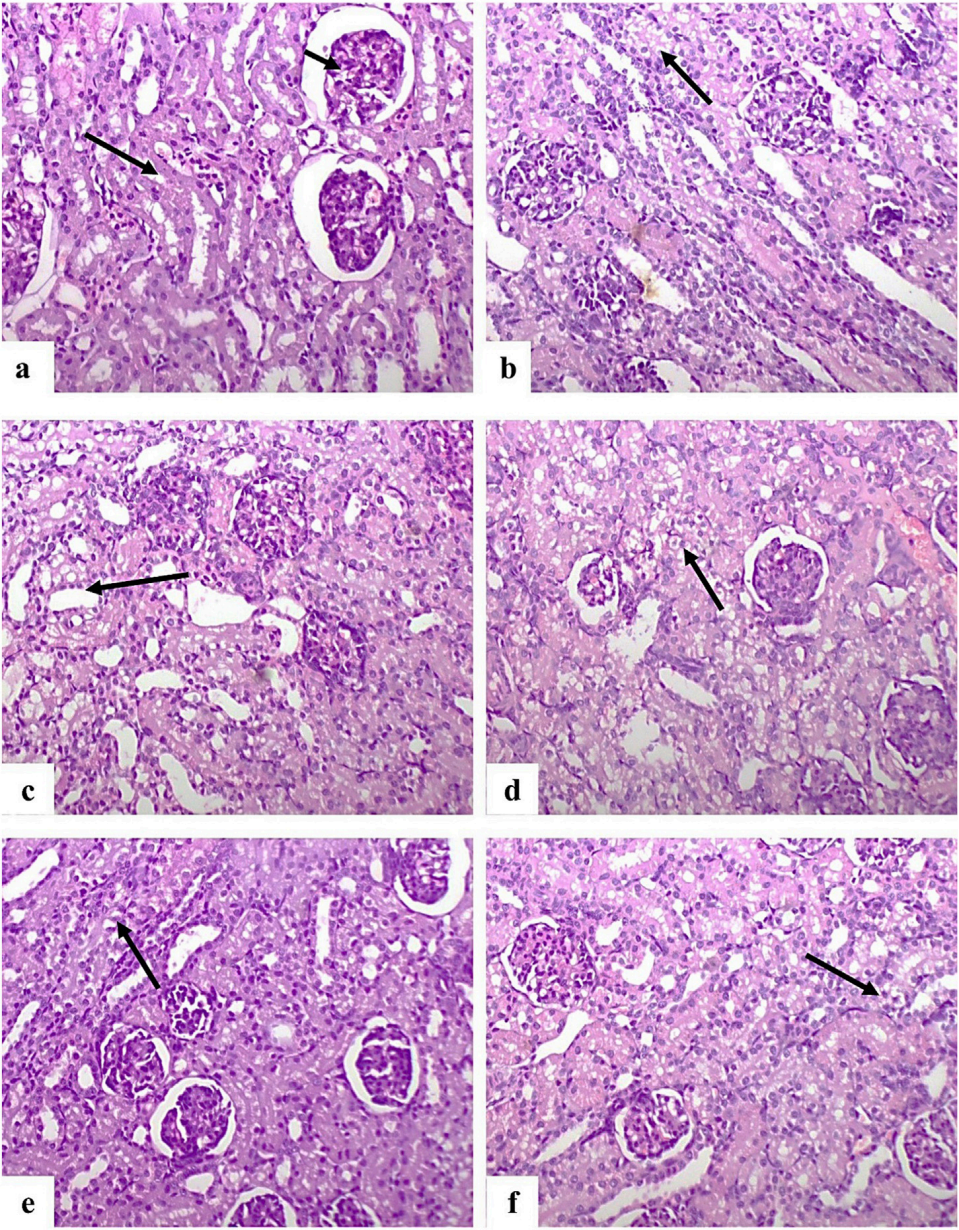
Photomicrographs of dog kidney sections stained with H&E (×200). **(a)** Control group showing normal renal tubules (long arrow) and corpuscles (short arrow). **(b,c)** G2 group showing vacuolar degeneration **(b)** and coagulative necrosis **(c)** of tubular epithelium (arrows). **(d)** G3 showing moderate vacuolar degeneration and necrosis (arrow). **(e)** G4 showing mild degeneration and necrosis (arrow). **(f)** G5 showing a few degenerated tubular epithelial cells (arrow).

In the kidney, normal histological structures of renal tubules and glomeruli were observed in the control group (Figure 6a). G2 showed prominent vacuolar degeneration and coagulative necrosis of the renal tubular epithelium (Figures 6b, c). Similar pathological changes were observed in G3 (Figure 6d), whereas G4 and G5 displayed only mild and minimal degenerative changes, respectively (Figures 6e, f).

**FIGURE 7:**
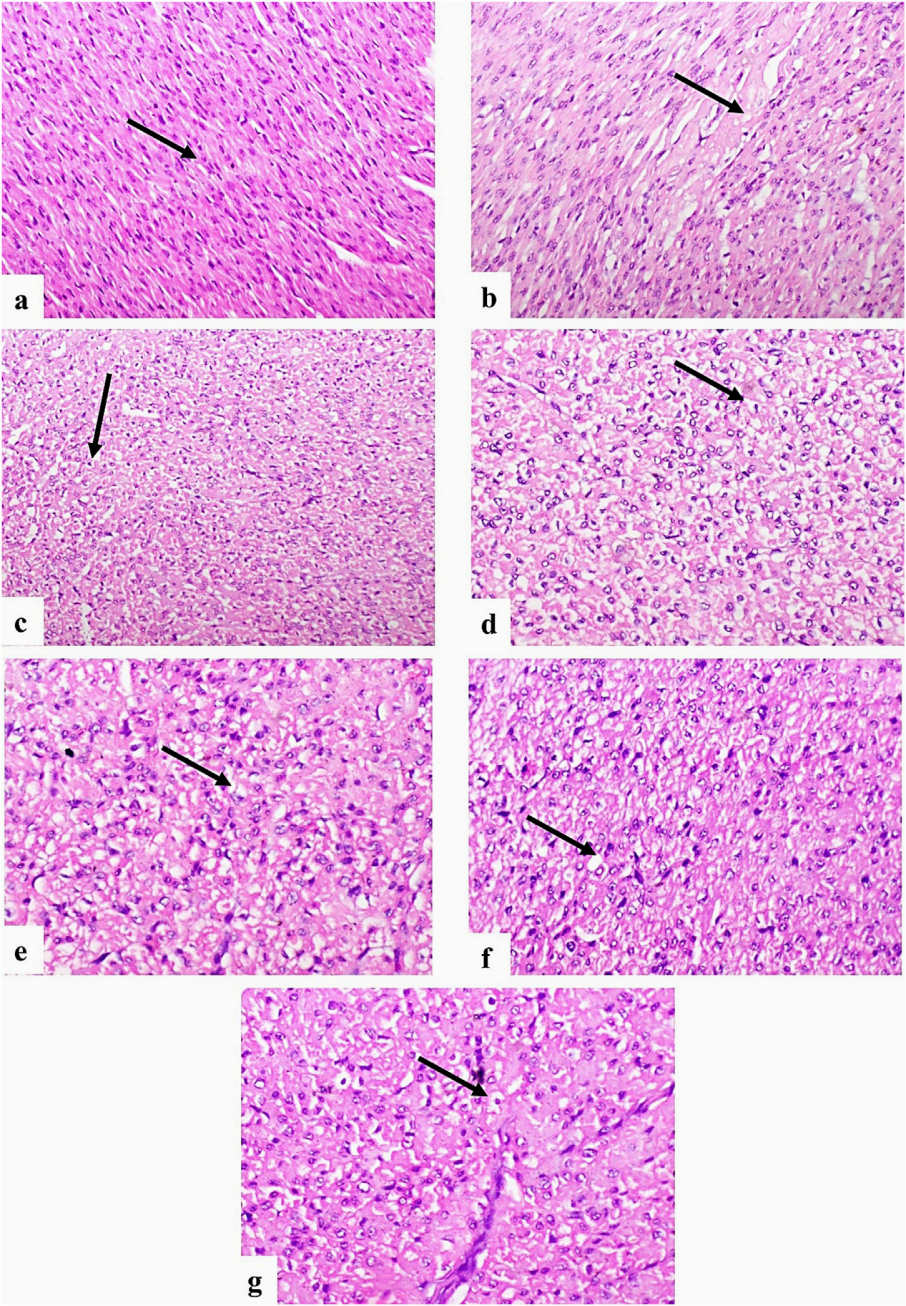
Photomicrographs of dog heart sections stained with H&E. **(a)** Control group showing normal myocyte structure (arrow) (×200). **(b,c)** G2 group showing Zenker’s necrosis **(b)** and diffuse vacuolation of myocytes **(c)** (arrows) (×200). **(d)** Higher magnification of **(c)** showing vacuolated myocytes (arrow) (×400). **(e)** G3 showing moderate vacuolar degeneration and Zenker’s necrosis (arrow) (×400). **(f)** G4 showing moderate vacuolation (arrow) (×400). **(g)** G5 showing mild degeneration and necrosis of myocytes (arrow) (×400).

Cardiac histology in the control group revealed normal myocardial fiber structure (Figure 7a). However, G2 showed Zenker’s necrosis and diffuse vacuolar degeneration (Figures 7b–d). G3 also exhibited vacuolar degeneration and Zenker’s necrosis (Figure 7e), while G4 displayed moderate vacuolation (Figure 7f), and G5 showed only mild degeneration and necrosis (Figure 7g). The severity of lesions in the liver, kidney, and heart was evaluated and scored, as presented in Table 6.

**TABLE 6:** Histopathological lesion scoring in liver, kidney and heart of treated groups.

Lesions	G1	G2	G3	G4	G5
Liver					
Degeneration of hepatocytes	0	3	2	1	1
Necrosis of hepatocytes	0	3	2	1	1
Portal fibrosis	0	2	1	0	0
Portal mononuclear inflammatory cells infiltration	0	2	1	0	0
Kidney					
Degeneration of renal tubular lining epithelium	0	3	2	1	1
Necrosis of renal tubular lining epithelium	0	3	2	1	0
Heart					
Degeneration of myocytes	0	3	2	1	1
Necrosis of myocytes	0	3	2	1	1

The score system was designed as: score 0 = absence of the lesion in all dogs of the group (n = 5), score 1= (<30%), score 2= (<30%–50%), score 3= (>50%).

## 4 Discussion

In the current study, we explored the therapeutic efficacy of tiamulin, bromhexine, and their combination in the management of experimentally induced *Staphylococcus* infections in dogs. Tiamulin, a pleuromutilin-class antimicrobial, exerts its bacteriostatic effect by selectively inhibiting protein synthesis through binding to the 50S ribosomal subunit, making it particularly effective against Gram-positive organisms, including *S. aureus* (Papich, 2016). However, the clinical success of antimicrobial therapy in staphylococcal infections often hinges not only on microbial susceptibility but also on adequate drug distribution to the site of infection. Bromhexine has been hypothesized to improve drug bioavailability and therapeutic outcomes (Matzembacker et al., 2024).

Our findings revealed that the combination of tiamulin and bromhexine led to a more pronounced clinical improvement and reduction in bacterial burden compared to either agent alone, suggesting a potential synergistic interaction. The dual therapy group (Tiamulin + Bromhexine) achieved the greatest bacterial reduction, indicating a synergistic or additive effect without evident antagonism. Recent veterinary research in bovines demonstrated that bromhexine hydrochloride can exert inherent antimicrobial activity and enhance the effect of other antibiotics, reinforcing its role as a viable adjunctive agent.

In preclinical studies, co-administration of bromhexine or its prodrug ambroxol with antibiotics has been shown to increase pulmonary antibiotic concentrations—most notably β-lactams and macrolides—though with variable clinical outcomes. One randomized clinical trial reported improved symptom resolution in respiratory infections treated with amoxicillin plus bromhexine versus amoxicillin alone. Nonetheless, concerns continue regarding inconsistencies in enhanced drug levels across antibiotic classes and infection sites (Deretic and Timmins, 2019). Our findings align with this positive trend, showing no diminution in tiamulin’s efficacy and instead suggest that bromhexine may enhance tissue penetration or distribution in canine *Staphylococcus* infections.

*In vitro* MIC testing in the present study showed that combining tiamulin with bromhexine led to a more pronounced reduction in bacterial growth than tiamulin alone, suggesting a potential enhancement in antibacterial activity. This observation may be explained by bromhexine’s known mucolytic properties, which can improve tissue diffusion and possibly alter bacterial membrane permeability. Earlier research demonstrated that bromhexine enhances antibiotic penetration into bronchial secretions (Matzembacker et al., 2024), and pharmacokinetic studies in poultry showed improved distribution of co-administered antimicrobials when combined with bromhexine (Ali et al., 2019). Although these effects were observed *in vitro* and may not fully translate to *in vivo* conditions, the reduced MIC values offer a rationale for further investigation into the potential synergistic role of bromhexine in antimicrobial protocols.

The observed superior efficacy of the dual therapy (tiamulin + bromhexine) extended beyond bacterial growth inhibition to a significant downregulation of key *S. aureus* virulence genes, such as *hla*, *ebpS*, and *icaA*. This downregulation implies that the combination therapy may impair pathogenic mechanisms such as α-hemolysin production (*hla*), fibronectin binding (*ebpS*), and PIA-mediated biofilm formation (*icaA*), thereby reducing bacterial virulence. The mechanism likely involves enhanced antibiotic penetration or disruption of bacterial regulatory pathways, possibly due to bromhexine’s mucolytic and membrane-modulating effects. The crucial roles of these genes are well established: *hla* encodes α-hemolysin, essential for host cell lysis (Lin et al., 2025); *icaA* is linked to biofilm matrix synthesis (Demir et al., 2020); and fibronectin-binding proteins like those encoded by *ebpS* are pivotal for tissue adhesion. Similar attenuation of virulence gene expression has been reported in studies involving antibiotic combinations targeting *S. aureus* and other Gram-positive pathogens. Thus, dual therapy not only reduces bacterial counts but also weakens pathogenic potential, making it a promising molecular-based strategy against *Staphylococcus* infections in dogs (Hammerschmidt et al., 2019).

In the present study, the blood parameters of the treated groups showed significant improvement compared to the *S. aureus*-infected control group. Infection with *S. aureus* is known to induce systemic inflammation, leading to hematological alterations such as leukocytosis, neutrophilia, lymphopenia, and anemia, as reported in previous studies (Kwiecinski and Horswill, 2020). These changes are attributed to the release of pro-inflammatory cytokines, oxidative stress, and bone marrow suppression caused by bacterial toxins. Our findings revealed that treated dogs exhibited restored levels of hemoglobin, red blood cells (RBCs), white blood cells (WBCs), and platelets compared to the infected untreated group. This suggests that the therapeutic interventions not only reduced bacterial burden but also alleviated the systemic effects of infection on hematopoiesis and immune response. Moreover, the recovery in leukocyte profiles in treated animals supports the hypothesis that the applied treatment protocols modulate the inflammatory response, reducing neutrophilic infiltration and restoring lymphocyte counts, consistent with findings from dog models (Farghali et al., 2019). Taken together, our data indicate that the tested therapeutic regimen provided effective control of the hematological alterations induced by *S. aureus* infection. Nevertheless, variations in response may depend on the treatment type, dosage, duration, and host factors. Further studies are warranted to elucidate the long-term impact of such treatments on hematological health and immune restoration.

In this study, dogs infected with *S. aureus* demonstrated a significant elevation in serum cardiac enzymes, including CK and CK-MB. These elevations suggest myocardial stress and possible subclinical cardiac involvement, likely resulting from systemic inflammatory responses and bacterial endotoxins (Janus et al., 2014). Following therapeutic intervention with tiamulin, bromhexine, or their combination, there was a marked reduction in the levels of these cardiac biomarkers. This improvement was most pronounced in the group receiving combined therapy, suggesting a synergistic cardioprotective effect. Tiamulin has been shown to effectively target Gram-positive organisms such as *S. aureus*, leading to reduced bacterial load and systemic inflammation (Xiang et al., 2022). In addition, bromhexine is known not only for its mucolytic action but also for enhancing tissue penetration of antibiotics and modulating immune responses, potentially reducing oxidative stress and protecting myocardial cells (Deretic and Timmins, 2019). The observed decrease in CK and CK-MB levels in the treated groups indicates a reduction in skeletal and myocardial muscle damage. Moreover, the superior outcomes in the group receiving both tiamulin and bromhexine may be attributed to improved drug delivery to inflamed tissues and enhanced host immune response. Although direct data on the cardiac effects of this combination are limited in canine models, similar therapeutic benefits have been reported in veterinary studies involving respiratory and systemic infections. To the best of our knowledge, there are currently no published studies in dogs evaluating the direct effects of bromhexine or tiamulin on serum cardiac troponin I levels. While absence of evidence is not evidence of absence, this notable gap highlights the originality of our findings.

In the present study, dogs infected with *S. aureus* exhibited a significant increase in liver enzymes ALT and AST. This elevation is consistent with previous reports indicating that systemic bacterial infections may lead to hepatic inflammation or dysfunction due to circulating endotoxins, oxidative stress, or immune-mediated hepatocellular damage (Murshed et al., 2025). The administration of tiamulin, bromhexine, or their combination showed a modulatory effect on these liver enzyme levels. Notably, the group treated with both agents demonstrated a more substantial decline in ALT and AST, suggesting an improved hepatic response. While tiamulin is generally regarded as safe for veterinary use, it has been reported to exhibit dose-dependent hepatotoxicity (Pankowska et al., 2024). Bromhexine, on the other hand, has shown hepatoprotective and antioxidant properties in various models, potentially by enhancing lysosomal enzyme release and reducing cellular inflammation (Radi et al., 2020). This may explain the more favorable liver enzyme profile in the group treated with bromhexine, particularly in combination with tiamulin. Overall, the reduction of liver enzyme levels in the treated groups may indicate not only clearance of infection but also a potential protective or stabilizing effect of the treatment protocols on hepatic tissues.

In this study, serum markers of renal function—urea, creatinine, and BUN—were significantly elevated in the *S. aureus*-infected group compared to the other groups. This suggests that the infection induced systemic effects extending to renal tissues, possibly through mechanisms such as sepsis-induced hypoperfusion, immune-mediated glomerular damage, or direct nephrotoxicity from bacterial toxins (Westgeest et al., 2022). According to Balkrishna et al. (2023), bacterial sepsis and endotoxemia are associated with impaired renal filtration and glomerular inflammation, leading to elevated serum urea and creatinine. The significant reduction of these renal markers in the treated groups, particularly in the combination therapy group, further supports the therapeutic benefit of bromhexine and tiamulin co-administration in mitigating renal damage during *S. aureus* infection.

Following therapeutic intervention with tiamulin, bromhexine, or their combination, there was a marked improvement in renal markers—especially in the combined treatment group—indicating a restoration of renal function and systemic stability. Although tiamulin is primarily excreted *via* the liver, its impact on kidney function has been shown to be minimal when used at therapeutic doses. Bromhexine, on the other hand, may indirectly support renal function through its anti-inflammatory and antioxidant actions, reducing systemic oxidative stress that can otherwise aggravate renal injury (Deretic and Timmins, 2019). The observed reduction in *urea*, *creatinine*, and *BUN* levels after treatment suggests that effective management of infection—especially using a combination of antimicrobial and supportive therapies—not only eradicates the pathogen but also contributes to the preservation or recovery of renal function.

The current findings revealed that infection with *S. aureus* led to a significant rise in MDA levels and a concurrent decline in TAC across the heart, liver, and kidney tissues of affected dogs. These alterations reflect the development of systemic oxidative stress and indicate that bacterial infection can compromise the oxidative balance in major organs. MDA is a key indicator of lipid peroxidation and is commonly used to assess cellular damage caused by oxidative stress. Its increased concentrations in these tissues suggest that *S. aureus* triggers organ-specific oxidative injury, likely through elevated reactive oxygen species (ROS) production, inflammatory responses, and toxin release from bacteria (Bastos et al., 2025). Simultaneously, the observed reduction in TAC highlights a weakened antioxidant defense, likely resulting from the rapid consumption of enzymatic and non-enzymatic antioxidants during the infection process. These patterns align with earlier research demonstrating similar oxidative stress responses in systemic bacterial conditions (Ponnampalam et al., 2022). After therapeutic intervention using tiamulin, bromhexine, or their combination, there was a noticeable improvement evidenced by reduced MDA levels and restored TAC. The most favorable outcomes were seen in the combined therapy group, indicating a potential synergistic effect. The role of bromhexine in this context may be attributed to its antioxidant and anti-inflammatory actions, including membrane stabilization and suppression of ROS generation (Bhagat, 2018). Meanwhile, tiamulin likely contributes indirectly by controlling bacterial growth and thereby minimizing oxidative burden.

Our findings showed that *S. aureus* infection significantly upregulated the expression of both *CYP1B1* and *IL-1β* genes, reflecting an enhanced oxidative and inflammatory response in the affected tissues. In the heart, *CYP1B1* is expressed in vascular endothelial cells, cardiomyocytes, and cardiac fibroblasts (Dubey et al., 2004) and is known to be highly inducible by oxidative stress. Its overexpression has been associated with inflammatory and immune responses. The observed upregulation of *CYP1B1* in infected dogs suggests that *S. aureus* triggered *ROS* production, leading to enhanced *CYP1B1* transcription. *CYP1B1* has been identified as a key mediator in angiotensin II-induced activation of NADPH oxidase, leading to *ROS* generation, and promoting vascular smooth muscle cell migration, proliferation, and hypertrophy (Malik et al., 2012). Studies in rats have demonstrated that elevated *CYP1B1* activity is linked to pathological conditions such as cardiac hypertrophy, vasoconstriction, endothelial dysfunction, and increased *ROS* production (Jennings et al., 2010; Jennings et al., 2014). *CYP1B1* enzymes can metabolize arachidonic acid and ω-3 polyunsaturated fatty acids (*PUFAs*) into medium-chain hydroxyeicosatetraenoic acids (*HETEs*), which possess cardiotoxic properties contributing to these adverse effects (Westphal et al., 2015). Conversely, *CYP1B1* inhibition has been shown to lower blood pressure and mitigate endothelial and renal dysfunction, as well as fibrosis, while simultaneously reducing *ROS* production (Gangadhariah et al., 2017; Jennings et al., 2014). Treatment with tiamulin and bromhexine, particularly in combination, resulted in a downregulation of *CYP1B1* expression, suggesting that the treatment effectively reduced oxidative stress. This may be attributed to bromhexine’s known antioxidant activity (Bhagat, 2018), along with the antimicrobial effect of tiamulin which suppresses the source of *ROS* stimulation. The specific impact of tiamulin on *CYP1B1* has not been extensively documented, but it is likely to be similar to its effects on other *CYP* isoforms, including inhibition (Pankowska et al., 2024).

*IL-1β* expression is a potent proinflammatory cytokine produced mainly by macrophages and neutrophils in response to microbial components, especially *via* Toll-like receptor (*TLR*) signaling. In our infected group, *IL-1β* expression was significantly elevated, consistent with activation of the innate immune response to bacterial invasion. Elevated *IL-1β* levels have been reported in numerous models of bacterial infection, including *S. aureus*, and are strongly linked to systemic inflammation and tissue damage (Rasquel-Oliveira et al., 2025). Therapeutic administration of tiamulin had an anti-inflammatory effect, which has been shown to suppress proinflammatory cytokine release (Xiang et al., 2022). In addition, therapeutic administration of bromhexine significantly downregulated *IL-1β* expression, particularly in the combined treatment group. This finding may reflect reduced bacterial load (Matzembacker et al., 2024) and the anti-inflammatory action of bromhexine, which has been shown to suppress proinflammatory cytokine release (Ansarin et al., 2020).

In our study, histopathological examination revealed significant structural alterations in the liver, kidney, and heart of the *S. aureus*-infected untreated group. The liver showed marked hepatocellular degeneration, congestion of central veins, and infiltration of inflammatory cells, indicating hepatic injury associated with infection. The kidneys displayed glomerular atrophy, tubular degeneration, and interstitial hemorrhage, reflecting renal damage. In the heart, myocardial fibers appeared disorganized with focal necrosis and leukocytic infiltration, suggesting cardiac involvement due to systemic infection (Onyebueke et al., 2018; Anton et al., 2024). In contrast, the tiamulin-treated group exhibited moderate improvement in tissue architecture. The hepatic and renal tissues showed reduced degeneration and inflammation compared to the untreated infected group, while the cardiac tissue displayed partial preservation of normal structure, although some pathological changes persisted. Bromhexine has mild effect on heart, liver, and kidney (El-Gendy et al., 2024b). This indicates a therapeutic effect of tiamulin in limiting bacterial-induced tissue damage. Remarkably, the group treated with the combination of tiamulin and bromhexine showed the most pronounced histological recovery. Nearly normal architecture was observed in the liver, kidney, and heart, with minimal inflammatory infiltration and absence of necrotic areas. The superior improvement may be attributed to the synergistic effect of tiamulin’s antibacterial action and bromhexine’s mucolytic and antioxidant properties, which may enhance drug absorption and tissue protection. These histological findings are consistent with the biochemical results, which demonstrated reduced oxidative stress markers (e.g., MDA) and improved antioxidant capacity (TAC), supporting the efficacy of the combined therapy in protecting vital organs against *S. aureus*-induced damage (Radi et al., 2020).

Overall, this study highlights that *S. aureus* infection leads to significant disruptions in the biochemical profile and histological integrity of critical organs such as the liver, kidneys, and heart. While tiamulin alone provided a moderate degree of protection, the co-administration of tiamulin and bromhexine offered superior therapeutic benefits. This was evident through improved antioxidant status and nearly normal tissue morphology. The enhanced efficacy of the combination suggests that bromhexine may serve as a valuable adjunct, boosting the performance of conventional antimicrobial therapy. Further investigations are recommended to explore the molecular basis of this synergism and assess the clinical relevance and long-term safety of such treatment strategies.

## 5 Conclusion

The present study confirms that *S*. *aureus* infection induces significant oxidative stress and multi-organ damage, as indicated by elevated MDA levels, reduced total TAC, and severe histopathological lesions in the liver, kidneys, and heart. Treatment with tiamulin alone resulted in moderate improvement in these parameters. However, the combined administration of tiamulin and bromhexine demonstrated superior therapeutic efficacy, as evidenced by a significant reduction in MDA levels, enhanced TAC activity, and notable restoration of normal tissue architecture. Additionally, the combination therapy achieved a lower MIC against *S. aureus*, significantly reduced bacterial loads in infected tissues, and markedly downregulated key virulence genes, including *hla*, *ica*, and *ebps*. These findings suggest that bromhexine enhances both the antibacterial and anti-virulence activities of tiamulin, providing a synergistic effect that may improve the management of *S. aureus*-induced infections. Further molecular and clinical investigations are warranted to validate these findings and assess the long-term safety and efficacy of this combinatorial approach.

## Data Availability

The original contributions presented in the study are included in the article/Supplementary Material, further inquiries can be directed to the corresponding author.
