# Supplementary material for: Bromhexine hydrochloride enhances the therapeutic efficacy of tiamulin against experimental Staphylococcus aureus infection in dogs: targeting bacterial virulence, boosting antioxidant defense, and improving histopathology
Source: Front Pharmacol. 2025 Dec 18;16:1679854. doi: 10.3389/fphar.2025.1679854 (PMC12756443; doi:10.3389/fphar.2025.1679854)
Supplement: Supplementary file 1 [file Supplementaryfile1.docx]

Supplementary Material

**Supplementary Table 1.** Biochemical identification of *Staph. aureus* obtained before induction of infection and after pyoderma-infected dogs.

| **Biochemical test** | ***Staphylococcus aureus* strain** |
| --- | --- |
| **Catalase** | + |
| **Coagulase** | + |
| **Alkaline phosphatase** | + |
| **Nitrate reduction** | + |
| **40% Bile Esculin** | - |
| **L-Pyrrolidonyl-B-naphthylamide** | - |
| **Mannitol fermentation** | + |
| **Urease activity** | - |
| **Mannose fermentation** | + |
| **Arginine reduction** | + |
| **Indoxyl phosphatase** | + |
| **Lactose fermentation** | + |
| **Novobiocin** | Resistant |

**Supplementary** **Table 2**. The experimental design.

|  |  | | **Day of sacrifice at 9^th^ day** | |
| --- | --- | --- | --- | --- |
| **Treatment**    **Groups** | **At 1^st^ day of the experiment** | **After 3 days of the experiment** | | **From 3^rd^ day till the end of the experiment**  **(for 5 days)** |
| **Group 1 (control negative)** | Normal saline (1ml intradermal) | No signs of infection | | Distilled water 1 ml (orally) |
| **Group 2 (control positive) (*Staph.aureus*)** | 10^5^ CFU *S. aureus*  (1ml intradermal) | Appear signs of infection | | Distilled water 1 ml (orally) |
| **Group 3**  **(Tiamulin+ *Staph.aureus*)** | 10^5^ CFU *S. aureus*  (1ml intradermal) | Appear signs of infection | | Tiamulin (orally)  10 mg/kg b.wt.  Once daily (every 24hrs) |
| **Group 4**  **(Bromhexine+ *Staph.aureus*)** | 10^5^ CFU *S. aureus*  (1ml intradermal) | Appear signs of infection | | Bromhexine (orally)  1mg/kg b.wt.  Twice daily(every 12hrs) |
| **Group 5 (Tiamulin+bromhexine+ *Staph.aureus*)** | 10^5^ CFU *S. aureus*  (1ml intradermal) | Appear signs of infection | | Tiamulin+bromhexine (orally)  10mg/kg b.wt. Once daily + 1mg/kg b.wt. Twice daily |

**Supplementary** **Table 3**. Primers sequence used in RT- PCR

| **Gene** | **Primer sequences** | **Annealing** | **Reference** |
| --- | --- | --- | --- |
| ***16S rRNA*** | F: 5′-CCTATAAGACTGGGATAACTTCGGG-3′ R: 5′-CTTTGAGTTTCAACCTTGCGGTCG-3′ | 55 ^o^C /30 sec. | **Mason et al., 2001** |
| ***icaA*** | F: 5′-CCTAACTAACGAAAGGTAG-3′  R: 5′-AAGATATAGCGATAAGTGC-3′ | 49 ^o^C /40 sec. | **Ciftci et al., 2009** |
| ***Ebps*** | F: 5′-AGAATGCTTTTGCAATGGAT-3′  R: 5′-AATATCGCTAATGCACCGAT-3′ | 50 ^o^C /30 sec. | **Vancra-eynest et al., 2004** |
| ***Hla*** | F: 5′-GAAGTCTGGTGAAAACCCTGA-3′ R: 5′-TGAATCCTGTCGCTAATGCC-3′ | 53 ^o^C /30 sec. | **Fei et al., 2011** |
| ***IL-1β*** NM_001037971 | F: 5′-TGCAAAACAGATGCGGATAA-3′  R: 5′-GTAACTTGCAGTCCACCGATT-3′ | 55 ^o^C /10 sec. | **Palaniappan et al., 2022** |
| ***CYP1B1*** MN_990717.1 | F: 5′-GCCGAGACGTTAACTACG -3′  R: 5′-CCTATTCCTACATGTAGG -3′ | 55 ^o^C /10 sec. | **Perepechaeva et al., 2024** |
| ***GAPDH*** XM_072783550.1 | F: 5′-AACATCATCCCTGCTTCCAC -3′  R: 5′-TCCTTGGAGGCCATGTAGAC -3′ | 55 ^o^C /10 sec. | **Aly et al., 2012** |

**Supplementary** **Table 4.** *In vitro* MIC values (μg/mL) of tiamulin, bromhexine hydrochloride, and combination of them against *Staph. aureus*

| **MIC (μg/mL)** | **Tiamulin** | **Bromhexine** | **Tiamulin + bromhexine** |
| --- | --- | --- | --- |
| ***Staph.aureus*** | 12.5 | 128 | 6.25+ 64 |
| **Serial Dilutions** | 0.7812 to 100 μg/mL | 16 to 2048 μg/mL | 0.7812 to 100 + 16 to 2048 μg/mL |

**2. Supplementary Figures**

**
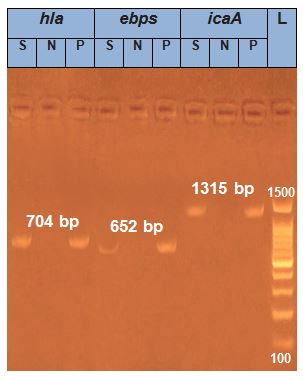
**

**Supplementary Figure 1:** Agarose gel electrophoresis of PCR amplification for ***hla*** (704 bp), ***ebpS*** (652 bp), and ***ica A*** (1315 bp) to identify virulence of *Staph. aureus*. L: 100 bp molecular weight ladder, S: sample, N: control negative, P: control positive.

**
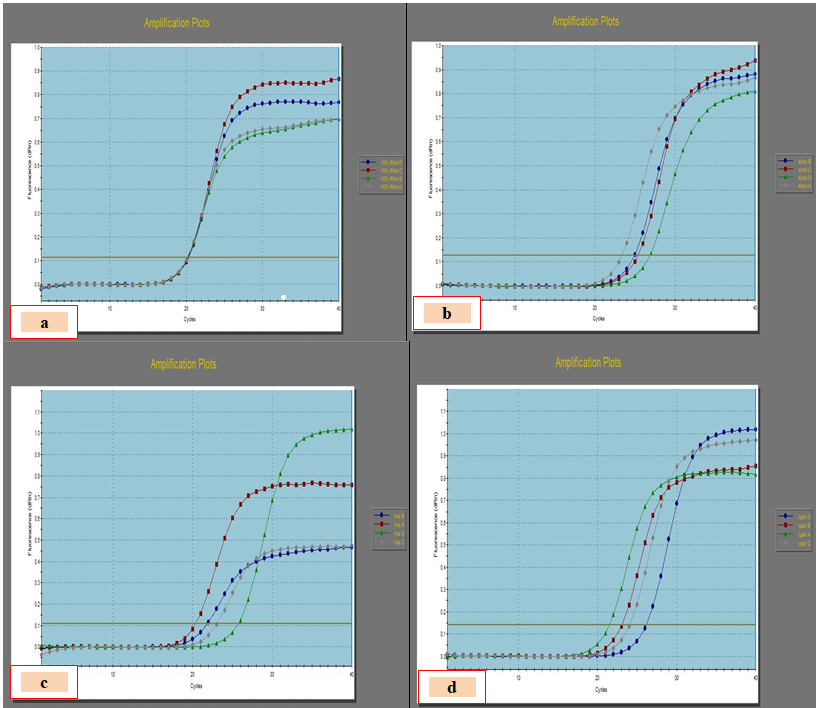
**

**Supplementary Figure 2:** Amplification curves representing gene-specific fluorescence during real-time PCR of *S. aureus* isolates. **a**; Fluorescence of *16S rRNA* gene, **b**; Fluorescence of *ebpS* gene, **c**; Fluorescence of *hla* gene, **d**; Fluorescence of *ica A* gene. A; Control group (*Staph. aureus*), B; Tiamulin+ *Staph. aureus*, C; Bromhexine+ *Staph. aureus*, D; Tiamulin+ Bromhexine+ *Staph. aureus*.
